# TRPML1 agonist ML‐SA5 attenuates pulmonary fibroblast activation by suppressing mTOR and restoring autophagic flux

**DOI:** 10.1002/2211-5463.70346

**Published:** 2026-09-24

**Authors:** Jiatong Yao, Xiaoting Kang, Lulu Meng, Shaoxuan Zhang, Qiujie Li, Jinyu Zhou, Mengyue Xue, Chu Li, Hua Zhu, Pengzhou Hang, Jing Zhao

**Affiliations:** ^1^ Department of Pharmacy Northern Jiangsu People's Hospital Yangzhou China; ^2^ Yangzhou Key Laboratory of Clinical Pharmacy and Drug Research Northern Jiangsu People's Hospital Affiliated to Yangzhou University Yangzhou China; ^3^ College of Pharmacy Dalian Medical University Dalian China

**Keywords:** autophagy, lung fibroblast, mTOR, pulmonary fibrosis, TRPML1

## Abstract

Modulating autophagic processes has shown promise as a treatment for pulmonary fibrosis (PF) with recent studies identifying transient receptor potential mucolipin‐1 (TRPML1), a lysosomal ion channel, as a novel regulator involved in PF. However, the mechanism by which TRPML1 modulates autophagy in the context of PF remains incompletely understood. Here we establish *in vitro* PF models by exposing human embryonic lung fibroblasts (MRC‐5) or primary lung fibroblasts to transforming growth factor (TGF‐β1). We then assessed the effects of the TRPML1 agonist ML‐SA5 on migratory capacity, the levels of fibrosis‐related proteins and autophagy markers, and autophagic flux. Our findings show that TGF‐β1 stimulation reduced TRPML1 protein levels, while ML‐SA5 treatment suppressed TGF‐β1‐induced cell migration, reduced the expression of fibrotic markers, increased LC3II levels, and restored autophagic flux in lung fibroblasts. Finally, investigating the role of mammalian target of rapamycin (mTOR) signaling in these effects using rapamycin (an mTOR inhibitor) and MHY1485 (an mTOR activator), we found ML‐SA5 inhibited mTOR signaling. Specifically, the anti‐fibrotic effects of ML‐SA5 were enhanced by co‐treatment with rapamycin and reversed by MHY1485. Thus, we conclude that ML‐SA5 attenuates TGF‐β1‐induced migration and collagen synthesis in pulmonary fibroblasts, at least in part through suppression of mTOR signaling and restoration of autophagic flux.

## Graphical abstract

TGF‐β1 stimulation downregulates lysosomal channel TRPML1 in pulmonary fibroblasts. The TRPML1 agonist ML‐SA5 suppressed fibroblast‐to‐myofibroblast activation and collagen production. Mechanistically, ML‐SA5 inhibited mTOR phosphorylation, restored autophagic flux, and its effects were enhanced by rapamycin (mTOR inhibitor) and reversed by MHY1485 (mTOR activator)—identifying the TRPML1–mTOR–autophagy axis as a potential therapeutic target in pulmonary fibrosis.

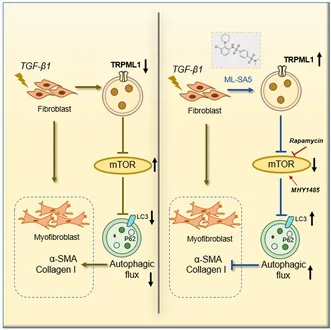

**Abbreviations**
AMPK: adenosine 5′‐monophosphate‐activated protein kinase
Col‐I: collagen I
CQ: chloroquine
CTGF: connective tissue growth factor
DMSO: dimethyl sulfoxide
ECM: extracellular matrix
FN: fibronectin
LC3: microtubule‐associated protein 1 light chain 3
mTOR: mammalian target of rapamycin
MTT: 3‐(4 5‐dimethylthiazol‐2‐yl)‐2,5‐diphenyl tetrazolium bromide
RAPA: rapamycin
STAT3: signal transducer and activator of transcription 3
TGF‐β1: transforming growth factor‐β1
TRPML1: transient receptor potential mucolipin 1
α‐SMA: α‐smooth muscle actin

## Introduction

Pulmonary fibrosis (PF) is a chronic progressive interstitial lung disorder defined by irreversible lung parenchymal fibrosis and aberrant lung tissue remodeling [1]. The core pathological event of PF is the aberrant activation of pulmonary fibroblasts, which undergo transdifferentiation into myofibroblasts that overproduce and secrete extracellular matrix (ECM) components including collagens, fibronectins, and elastins. This process ultimately disrupts normal alveolar structure and compromises gas exchange [2, 3]. Patients with PF have a poor prognosis, with a median survival of only 3–5 years after diagnosis [4]. Although clinical anti‐fibrotic drugs like pirfenidone and nintedanib can slow down the progressive course of PF, they cannot reverse existing fibrotic lesions or target the fundamental pathophysiological mechanisms, and their clinical utility is limited by adverse effects and variable efficacy [5]. Therefore, identifying molecular targets capable of halting or reversing fibrogenesis remains an urgent priority.

Autophagy is a highly evolutionarily conserved cellular process responsible for eliminating misfolded proteins and damaged organelles to maintain homeostasis. Emerging evidence implicates autophagic dysfunction in the pathogenesis of various fibrotic disorders, including PF [6, 7, 8]. However, the precise relationship between autophagy and lung fibrogenesis remains incompletely defined [9, 10, 11]. Consequently, strategies aimed at restoring autophagic flux have been proposed as potential therapeutic approaches for PF.

Transient receptor potential mucolipin 1 (TRPML1) is a non‐selective cation channel primarily distributed on the membranes of late endosomes and lysosomes, which mediates the release of Ca^2+^ from lysosomes to the cytoplasm [12]. TRPML1 activity influences lysosomal biogenesis and regulates transcription of autophagy‐related genes [13], and the channel directly participates in autophagosome–lysosome fusion [14]. Dysfunction of TRPML1 has been implicated in multiple pathologies [15, 16], and recent studies by Weiden et al. have identified TRPML1 as a potential therapeutic target in PF [17]. Notably, they demonstrated that TRPML1 suppresses PF by limiting collagen and elastin deposition and reported that TRPML1 agonists exert anti‐fibrotic effects by increasing matrix metalloproteinase levels. Meanwhile, they also found that autophagy was reduced in TRPML1‐deficient lung fibroblasts [17]. However, the precise signaling mechanisms linking TRPML1 activation to autophagy restoration in fibroblasts, particularly the involvement of mTOR signaling, remain incompletely understood. ML‐SA5 is a potent, selective small‐molecule agonist of TRPML1 widely employed to probe channel function and its physiological relevance [18, 19, 20].

In the present study, we developed an *in vitro* cell model of fibroblast activation induced by TGF‐β1 using human embryonic lung fibroblasts (MRC‐5) and rat primary pulmonary fibroblasts. We investigated whether pharmacologic activation of TRPML1 with ML‐SA5 modulates fibroblast activation and migratory capacity, and examined the contribution of mTOR‐regulated autophagy to the observed anti‐fibrotic effects. Our objectives were to elucidate TRPML1's role in fibroblast biology and to assess the therapeutic potential of ML‐SA5 in PF.

## Materials and methods

### Reagents

TRPML1 agonist ML‐SA5 (HY‐152182), antagonist ML‐SI3 (HY‐139426), rapamycin (RAPA, HY‐10219), and mTOR agonist MHY1485 (HY‐B0795) were purchased from MedChemExpress (Shanghai, China). Recombinant human transforming growth factor (TGF‐β1, 10 804‐HNAC) was obtained from Sino Biological (Beijing, China). Primary antibodies were obtained from the following suppliers: TRPML1 (ACC‐081) was purchased from Alomone Labs. Collagen type I (Col‐I, 14695‐1‐AP) and connective tissue growth factor (CTGF, 23936‐1‐AP) were supplied by Proteintech. Fibronectin (FN, sc‐8422) was obtained from Santa Cruz Biotechnology, while Beclin‐1 (A0562) was from ABclonal. LC3 (ab192890) and p62 (ab109012) were acquired from Abcam. The remaining primary antibodies, including α‐smooth muscle actin (α‐SMA, 19245), TGF‐β1 (3711), p‐Smad2/3 (8828), Smad2/3 (8685), p‐STAT3 (Tyr705, 9131), STAT3 (4904), p‐AKT (Ser473, 4060), AKT (4691), p‐AMPK (Thr172, 2535), AMPK (2532), p‐mTOR (Ser2448, 2971), and mTOR (2983), were all purchased from Cell Signaling Technology.

### Cell culture and treatment

Human embryonic lung fibroblast cell line MRC‐5 (RRID:CVCL_0440) was obtained from Procell Life Science & Technology Co., Ltd. (Wuhan, China) and authenticated by the supplier upon acquisition. Master stocks were established immediately after receipt and cryopreserved in liquid nitrogen. All experiments were performed using mycoplasma‐free cells thawed from these original frozen stocks within the past 3 years. The cells (passage 5–15) were cultured in Minimum Essential Medium (MEM) containing 10% fetal bovine serum and 1% penicillin–streptomycin, and incubated at 37 °C in a humidified incubator with 5% CO_2_. Eight experimental groups were established: control (Ctrl), TGF‐β1 alone (10 ng/mL), TGF‐β1 combined with ML‐SA5 (1, 5, or 10 μm), and ML‐SA5 alone (1, 5, or 10 μm). ML‐SA5 was added 30 min prior to TGF‐β1 stimulation, and cells were harvested after 48 h. To assess autophagy involvement, cells were treated with the mTOR inhibitor RAPA (10 μm) either alone or together with ML‐SA5. Drug concentrations and treatment durations were selected based on preliminary trials and published reports. For mTOR pathway studies, cells were exposed to RAPA or MHY1485 following previously described protocols [21].

### Isolation and culture of rat primary lung fibroblasts and adult mouse lung fibroblasts

All animals were maintained under standard laboratory conditions (25 °C, 40%–60% relative humidity, 12‐h light/12‐h dark cycle) with *ad libitum* access to standard rodent chow and fresh water. The study was reported following the ARRIVE 2.0 guidelines. All animal procedures were approved by the Institutional Animal Care and Use Committee of Yangzhou University (approval No. 202402859) and conducted in accordance with the well‐established experimental protocols [22, 23]. Primary rat lung fibroblasts (RPLFs) were isolated from 1 to 3‐day‐old Wistar rats of both sexes. Briefly, the lung tissues of the rats were excised, rinsed thoroughly with ice‐cold phosphate‐buffered saline (PBS), and cut into small fragments with a volume of approximately 1–2 mm^3^. The tissue fragments were then digested with 0.25% trypsin–EDTA under gentle shaking conditions. The resulting suspension was passed through a 70‐μm cell strainer and centrifuged at 160 *g* for 10 min at 4 °C. Pelleted cells were resuspended in DMEM containing 10% FBS and 1% penicillin–streptomycin and then seeded into culture flasks and maintained at 37 °C with 5% CO_2_. At 80%–90% cellular confluence, the fibroblasts were passaged and exposed to identical treatment conditions as applied to MRC‐5 cells.

Adult mouse lung fibroblasts (AMLFs) were cultured according to previous studies [24]. Male C57BL/6 mice (8–10 weeks old) were deeply anesthetized via intraperitoneal injection of tribromoethanol (Avertin®; Sigma‐Aldrich, 200 mg/kg, dissolved in tert‐amyl alcohol and diluted in saline). After the loss of the pedal withdrawal reflex was confirmed, the mice were euthanized by cervical dislocation while under deep anesthesia. A thoracotomy was immediately performed to expose the heart and lungs. To remove blood contaminants, the lungs were perfused *in situ* via the right ventricle with ice‐cold sterile PBS until the pulmonary vasculature was fully blanched. The entire lung lobes were aseptically harvested, placed in ice‐cold sterile HBSS, followed by repeated rinsing to eliminate blood and debris. The tissue was minced into ~1 mm^3^ fragments and digested with 1 mg/mL collagenase II at 37 °C for 30 min. The cell suspension was filtered through a 70 μm cell strainer, centrifuged at 1000 rpm for 5 min, and the pellet was resuspended in DMEM complete medium and seeded into culture flasks at 37 °C in 5% CO_2_. When cultures reached 70%–80% confluence, cells were passaged using 0.25% trypsin, and passages 3–6 were used for all experiments.

### Live‐cell morphology staining

The cells were seeded in 6‐well culture plates at a seeding density of 1 × 10^5^ cells per well. Upon reaching approximately 50% confluence, cells were subjected to the indicated treatments. After that, the culture medium was discarded, and the cells were incubated with 2 μm Calcein‐AM (Thermo, USA) at 37 °C for 15 min in a light‐protected environment, followed by two washes with PBS. Fluorescence images were obtained via an Olympus IX83 inverted fluorescence microscope (Olympus, Tokyo, Japan) for the assessment of cell morphological features and viability status.

### Wound healing assay

Cell migration was assessed using a scratch wound‐healing assay as described previously [25]. Cells were seeded in 6‐well plates at a high density (3 × 10^5^ cells/well). After forming a confluent monolayer, a sterile 200‐μL pipette tip was used to create a uniform scratch (~500 μm width). Debris was removed by two PBS washes, and the medium was replaced with serum‐free MEM containing the respective treatments to minimize proliferation effects. Phase‐contrast images were taken at 0 and 48 h post‐scratch using an Olympus IX83 microscope. The wound area of each group was quantified using Image‐Pro Plus 5.0 software (Media Cybernetics, Rockville, MD, USA), and the cell migration area was calculated by subtracting the wound area at 48 h from the initial wound area at 0 h.

### mCherry‐GFP‐LC3 staining

mCherry‐GFP assay was used to evaluate the autophagy flux efficiency according to previous studies [26]. In autophagosomes (neutral pH ~7.4), both GFP and mCherry fluoresce, producing yellow puncta (GFP^+^/mCherry^+^). Upon fusion with lysosomes, the autophagosome lumen becomes acidic (pH ~4.5–5.0), which quenches GFP fluorescence while mCherry fluorescence persists, producing red puncta (GFP^−^/mCherry^+^). Therefore, yellow puncta represent autophagosomes (early autophagic structures), red puncta represent autolysosomes (late autophagic structures indicating successful fusion), and the red: yellow puncta ratio reflects autophagic flux efficiency. Approximately 1 × 10^5^ MRC‐5 cells per dish were seeded into confocal dishes and incubated overnight. At ~50% confluence, cells were transduced with mCherry‐GFP‐LC3 adenovirus vectors (Beyotime, China) at an MOI of 2 [27]. Following a 12‐h incubation, the virus‐laden medium was replaced with freshly prepared complete culture medium. A positive control group received 20 μm chloroquine (CQ) for 24 h to block autophagic flux. Following the completion of treatment, the cells were washed thoroughly with PBS, and live‐cell imaging assays were performed utilizing an Olympus IX83 microscope. Autophagosomes (yellow puncta: GFP^+^/mCherry^+^) and autolysosomes (red puncta: GFP^−^/mCherry^+^) were counted using Image‐Pro Plus software.

### Intracellular calcium concentration determination

RPLFs were incubated with 5 μm Fluo‐8/AM dye at 37 °C in the dark for 40 min to detect calcium signaling as described previously [28], and the baseline fluorescence intensity was recorded. Subsequently, the fluorescence intensity was measured after 10 min of ML‐SA5 (1 μm) treatment, followed by a further 10 min of incubation with ML‐SI3 (2.5 μm), and the corresponding fluorescence intensity was continuously monitored. The fluorescence intensity values of 30 individual cells were quantified for analysis.

### Western blotting

Total protein extracts from the cells were obtained using RIPA lysis buffer containing PMSF (Beyotime, Haimen, China) and phosphatase inhibitors (Roche, Germany). Protein concentrations were determined using the bicinchoninic acid (BCA) assay (Beyotime, Shanghai, China) according to the manufacturer's instructions, and equal amounts (70–100 μg per lane) were resolved by 8–12% SDS/PAGE and transferred onto nitrocellulose membranes. Membranes were blocked with 5% nonfat milk in PBS for 2 h at room temperature, then incubated overnight at 4 °C with primary antibodies. After washing with PBST, membranes were incubated with IRDye 800CW‐conjugated secondary antibodies (LI‐COR Biosciences, Lincoln, NE, USA; 1 : 10000) for 1 h at room temperature in the dark. Bands were detected utilizing an Odyssey CLx infrared imaging system (LI‐COR Biosciences), and densitometric analysis was performed with Image Studio Lite software (version 5.2, LI‐COR Biosciences). GAPDH or β‐actin served as loading controls for normalization.

### Immunofluorescence

Following treatment, cells were fixed with 4% paraformaldehyde for 15 min, subsequently washed, and then permeabilized with 0.02% Triton X‐100. After blocking with 10% goat serum at room temperature, cells were incubated with primary antibodies overnight at 4 °C. Subsequently, the corresponding fluorescent secondary antibodies (Dylight 488 or Dylight 594) were applied and incubated for 1 h at room temperature in the dark. Nuclei were counterstained with Hoechst dye for 20 min. For fluorescence quantification, images were acquired using an Olympus IX83 fluorescence microscope with consistent exposure settings across all groups. The microscope focus was determined using the Hoechst nuclear counterstain as a reference plane, with the focal plane set at the maximal nuclear cross‐sectional area to ensure consistency. For each group, six random high‐power fields were selected from different regions of the slide to avoid selection bias. Region of interest (ROI) was manually drawn around individual cells using the freehand selection tool in Image‐Pro Plus 5.0, with cell boundaries defined by the Hoechst nuclear staining and the corresponding phase‐contrast image. Only cells with clearly defined borders and intact morphology were included in the analysis.

### Statistical analysis

All experimental data were expressed as Mean ± SEM, and statistical analyses were conducted using graphpad Prism 8.0 software (graphpad Software, Inc., CA, USA). For western blot data analysis, we normalized the data to the internal control within each individual batch and subsequently analyzed the normalized values collectively. The normality of all datasets was assessed using the Shapiro–Wilk's test. For parametric data that were normally distributed, statistical significance of differences was assessed using an unpaired two‐tailed Student's *t*‐test for two‐group comparisons, and by one‐way ANOVA followed by Tukey's *post hoc* test for multiple‐group comparisons. For nonparametric data, the Mann–Whitney *U*‐test and Kruskal–Wallis test for one‐way ANOVA followed by the Dunn *post hoc* test were performed for comparisons between two groups. A two‐tailed *P* value < 0.05 was considered statistically significant.

## Results

### ML‐SA5 attenuates TGF‐β1‐induced activation of MRC‐5 cells

**Fig. 1:**
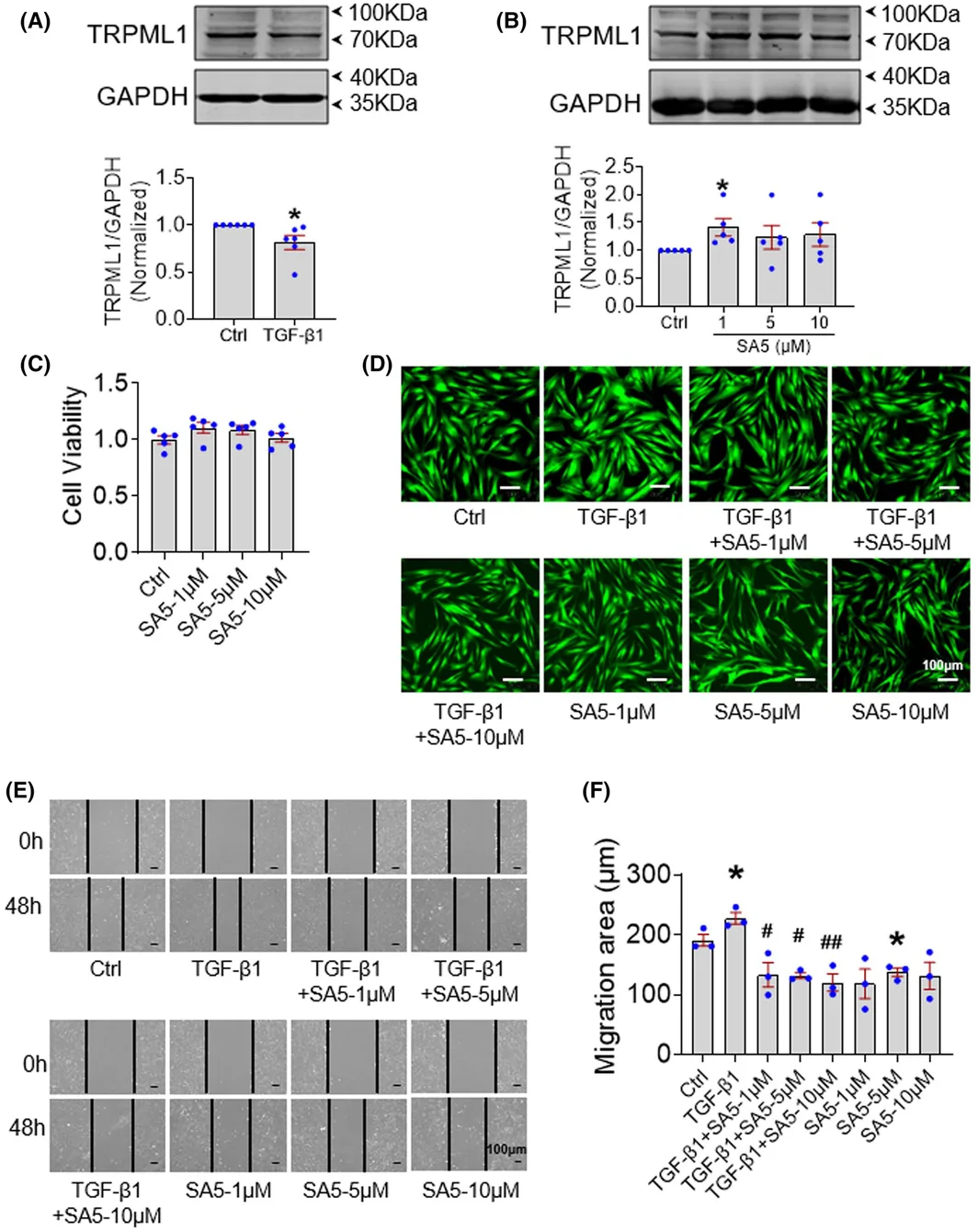
ML‐SA5 attenuates transforming growth factor‐β1 (TGF‐β1)‐induced activation of MRC‐5 cells. (A) Representative western blot images and quantitative analysis of transient receptor potential mucolipin 1 (TRPML1) protein expression in MRC‐5 cells subjected to the pulmonary fibrosis model induced by TGF‐β1 (10 ng/mL) for 48 h. *P < 0.05 vs. Ctrl, *n* = 6. (B) Representative western blot bands and statistical results of TRPML1 channel expression in MRC‐5 cells treated with varying concentrations of the small‐molecule agonist ML‐SA5 (1 μm, 5 μm, 10 μm). *P < 0.05 vs. Ctrl, *n* = 5. (C) Cell viability of MRC‐5 cells treated with ML‐SA5, *n* = 5. (D) Morphological changes in MRC‐5 cells from each group observed under a fluorescence microscope following Calcein‐AM staining. MRC‐5 cells were divided into eight groups: control (Ctrl), TGF‐β1 alone (10 ng/mL), TGF‐β1 combined with ML‐SA5 (1, 5, or 10 μm), and ML‐SA5 alone (1, 5, or 10 μm). ML‐SA5 was added 30 min prior to TGF‐β1 stimulation, and cells were harvested after 48 h. (E, F) Representative images of wound‐healing assays in MRC‐5 cells at 0 and 48 h, along with quantitative analysis of the migration distance. *P < 0.05 vs. Ctrl; #*P* < 0.05, ##*P* < 0.01 vs. TGF‐β1, *n* = 3. Data are presented as mean ± SEM. Mann–Whitney test, one‐way ANOVA followed by Tukey's *post hoc* test, and Kruskal–Wallis test for one‐way ANOVA followed by the Dunn *post hoc* test were used for statistical analysis.

To assess the role of TRPML1 in fibroblast activation, we established an *in vitro* model using TGF‐β1‐stimulated MRC‐5 cells. Western blot assay results showed that TRPML1 protein expression was significantly downregulated in MRC‐5 cells after TGF‐β1 stimulation (Fig. 1A). Dose–response experiments showed that ML‐SA5 at 1, 5, and 10 μm significantly restored TRPML1 expression, with 1 μm producing the most robust effect (Fig. 1B). MTT assays confirmed that ML‐SA5 at concentrations up to 10 μm exhibited no cytotoxicity (Fig. 1C). Calcein‐AM staining demonstrated that TGF‐β1 induced characteristic morphological changes associated with fibroblast activation, including increased cell density and elongated, enlarged cellular morphology. These alterations were attenuated by ML‐SA5 treatment (Fig. 1D). Wound‐healing assays demonstrated that TGF‐β1 treatment significantly enhanced the migratory capacity of MRC‐5 cells compared with untreated control cells. In contrast, ML‐SA5 markedly attenuated this TGF‐β1‐induced cell migration, with significant inhibitory effects detected at a concentration of 1 μm (Fig. 1E,F).

### ML‐SA5 modulates TGF‐β1‐induced expression of fibrosis‐associated molecules

**Fig. 2:**
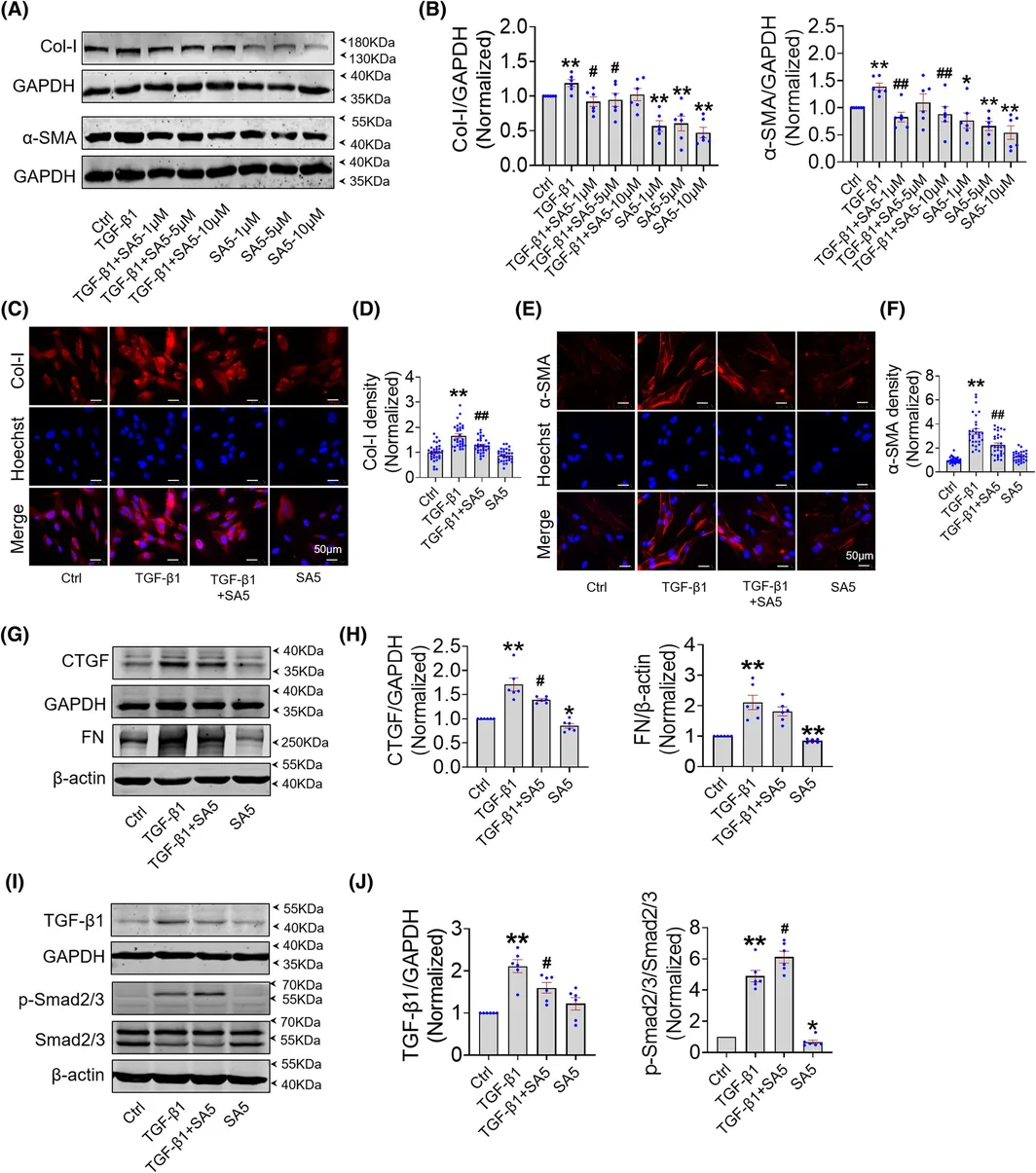
Ml‐SA5 modulates transforming growth factor‐β1 (TGF‐β1)‐induced expression of fibrosis‐associated molecules. (A, B) Protein expression levels and quantitative analysis of extracellular matrix component collagen I (Col‐I) and transdifferentiation marker α‐smooth muscle Actin (α‐SMA) in MRC‐5 cells stimulated with TGF‐β1 (10 ng/mL) for 48 h in the presence or absence of varying concentrations of ML‐SA5 (1 μm, 5 μm, 10 μm). *P < 0.05, **P < 0.01 vs. Ctrl; #*P* < 0.05, ##*P* < 0.01 vs. TGF‐β1, *n* = 6. (C, D) Representative immunofluorescence staining images and quantitative analysis of Col‐I in MRC‐5 cells. Red fluorescence indicates Col‐I, and blue fluorescence indicates cell nuclei. Scale bar: 50 μm. **P < 0.01 vs. Ctrl; ##*P* < 0.01 vs. TGF‐β1, *n* = 30 cells from 6 independent biological replicates. (E, F) Representative immunofluorescence staining images and quantitative analysis of α‐SMA in MRC‐5 cells. Red fluorescence indicates α‐SMA, and blue fluorescence indicates cell nuclei. Scale bar: 50 μm. **P < 0.01 vs. Ctrl; ##*P* < 0.01 vs. TGF‐β1, *n* = 30 cells from 6 independent biological replicates. (G, H) Representative western blot images and quantitative analysis of connective tissue growth factor (CTGF) and fibronectin (FN) protein expression in MRC‐5 cells. *P < 0.05, **P < 0.01 vs. Ctrl; #*P* < 0.05 vs. TGF‐β1, *n* = 6. (I, J) Representative western blot bands and statistical results of TGF‐β1 and Smad2/3 protein expression in MRC‐5 cells. *P < 0.05, **P < 0.01 vs. Ctrl; #*P* < 0.05 vs. TGF‐β1, *n* = 6. Data are presented as mean ± SEM. One‐way ANOVA followed by Tukey's *post hoc* test and Kruskal–Wallis test for one‐way ANOVA followed by the Dunn *post hoc* test were used for statistical analysis.

Given that excessive extracellular matrix deposition and fibroblast‐to‐myofibroblast transdifferentiation are hallmarks of PF, we examined expression of Col‐I and α‐SMA. TGF‐β1 stimulation substantially elevated Col‐I production and α‐SMA expression, whereas co‐treatment with ML‐SA5 dose‐dependently attenuated these fibrotic markers. Notably, 1 μm ML‐SA5 significantly reduced Col‐I accumulation and inhibited α‐SMA induction (Fig. 2A,B). Immunofluorescence staining corroborated these findings, revealing reduced collagen deposition and fewer α‐SMA‐positive cells following treatment with 1 μm ML‐SA5 (Fig. 2C–F). Critically, we have also performed live‐cell Ca^2+^ imaging to directly confirm that our agonists indeed activate TRPML1‐mediated calcium signaling in treated cells. Using the fluorescent calcium indicator Fluo‐8/AM, we monitored intracellular Ca^2+^ oscillations in primary lung fibroblasts following agonist application. We observed a robust and reproducible increase in cytosolic Ca^2+^ levels upon agonist treatment, which was significantly blunted by pre‐incubation with the TRPML1 inhibitor ML‐SI3 (Fig. S1). Furthermore, TGF‐β1 stimulation markedly upregulated CTGF, FN, and TGF‐β1 itself in MRC‐5 cells. Co‐treatment with 1 μm ML‐SA5 significantly reduced CTGF and TGF‐β1 protein expression (Fig. 2G–J); however, ML‐SA5 did not significantly suppress FN expression. Notably, ML‐SA5 had no appreciable effect on Smad2/3 phosphorylation, a canonical downstream effector of TGF‐β1 signaling (Fig. 2I,J), suggesting that its anti‐fibrotic effects occur through Smad‐independent mechanisms.

### ML‐SA5 restores autophagic flux in TGF‐β1‐treated MRC‐5 cells

**Fig. 3:**
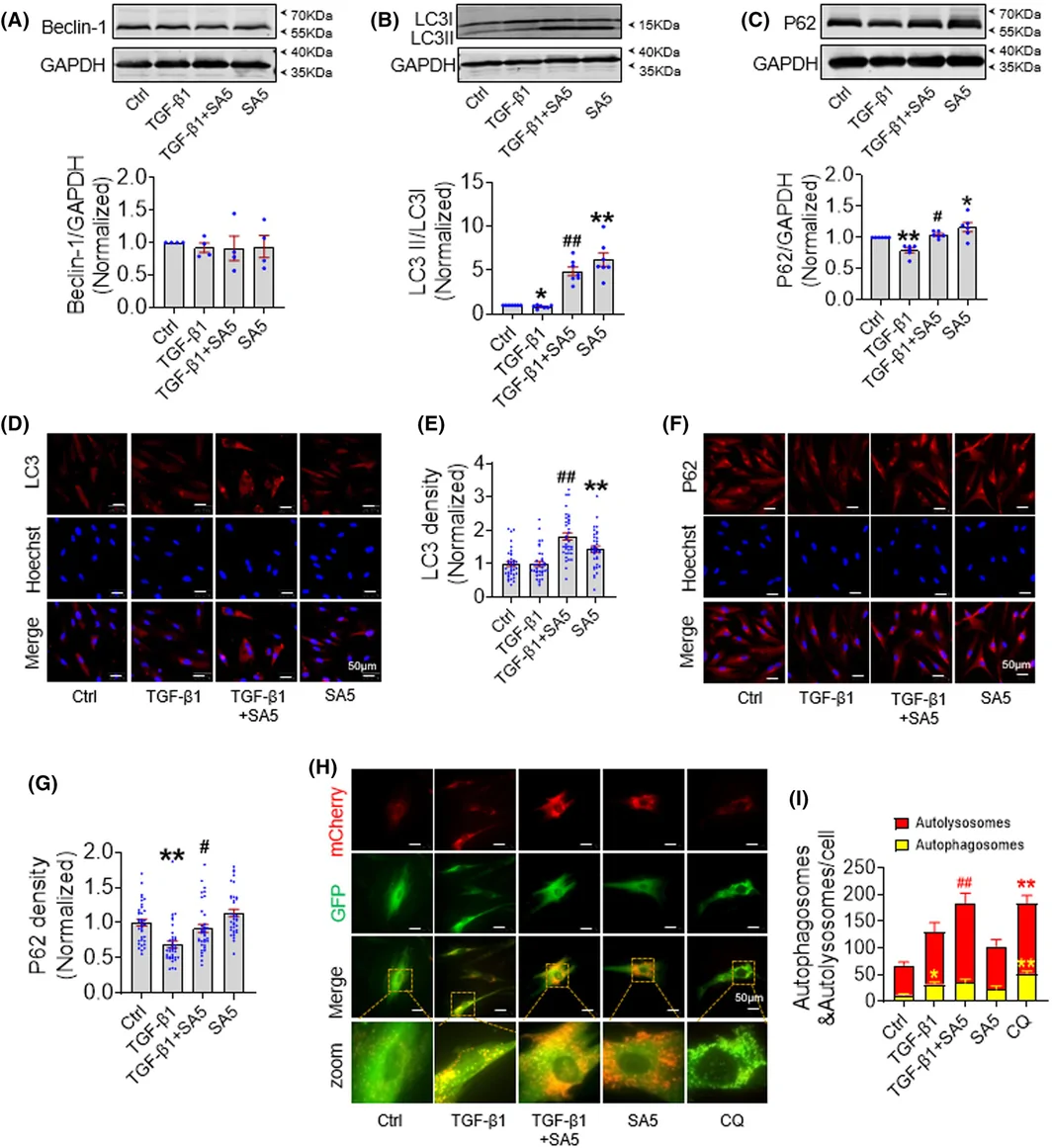
ML‐SA5 restores autophagic flux in transforming growth factor‐β1 (TGF‐β1)‐treated MRC‐5 cells. MRC‐5 cells were divided into four groups: control (Ctrl), TGF‐β1 alone (10 ng/mL), TGF‐β1 combined with ML‐SA5 (1 μm), ML‐SA5 alone, respectively. For the combination group, ML‐SA5 was administered 30 min before TGF‐β1 stimulation, and all cells were collected 48 h post‐treatment. (A) Representative western blot images and quantitative analysis of Beclin‐1 protein expression in MRC‐5 cells, *n* = 4. (B) Representative western blot images and quantitative analysis of LC3 protein expression in MRC‐5 cells, *n* = 7. *P < 0.05, **P < 0.01 vs. Ctrl; ##*P* < 0.01 vs. TGF‐β1. (C) Representative western blot images and quantitative analysis of p62 protein expression in MRC‐5 cells, *n* = 6. *P < 0.05, **P < 0.01 vs. Ctrl; #*P* < 0.05 vs. TGF‐β1. (D, E) Representative images and quantitative analysis of LC3 immunofluorescence staining. Scale bar: 50 μm. **P < 0.01 vs. Ctrl; ##*P* < 0.01 vs. TGF‐β1, *n* = 36 cells from 6 independent biological replicates. (F, G) Representative images and quantitative analysis of p62 immunofluorescence staining. Scale bar: 50 μm. **P < 0.01 vs. Ctrl; #*P* < 0.05 vs. TGF‐β1, *n* = 36 cells from 6 independent biological replicates. (H, I) Representative images and quantification of autophagic flux regulation in MRC‐5 cells treated with ML‐SA5. Chloroquine (CQ, 20 μm) was used as a positive control. Scale bar: 50 μm. **P < 0.01 vs. Ctrl; ##*P* < 0.01 vs. TGF‐β1, *n* = 20. Data are presented as mean ± SEM. One‐way ANOVA followed by Tukey's *post hoc* test and Kruskal–Wallis test for one‐way ANOVA followed by the Dunn *post hoc* test were used for statistical analysis.

To determine whether ML‐SA5 modulates autophagy, we evaluated autophagy‐related proteins and flux dynamics. ML‐SA5 did not significantly alter total Beclin‐1 levels (Fig. 3A). The LC3II/LC3I ratio and p62 expression were decreased by TGF‐β1; however, ML‐SA5 treatment restored both the LC3II/LC3I ratio and p62 levels compared with the TGF‐β1 group (Fig. 3B,C). Immunofluorescence revealed increased LC3 and p62 puncta following ML‐SA5 treatment (Fig. 3D–G). Using the mCherry‐GFP‐LC3 tandem reporter assay, we observed that TGF‐β1 increased yellow (GFP^+^mCherry^+^) puncta—indicative of autophagosomes—and decreased the red: yellow puncta ratio, consistent with impaired autophagosome–lysosome fusion. ML‐SA5 increased both green and yellow signals, indicating enhanced autophagosome formation, and increased red puncta together with the red:yellow ratio, reflecting increased autolysosomes. To unequivocally demonstrate that the elevated p62 observed with ML‐SA5 does not indicate a block in autophagic degradation, we further performed additional western blot experiments using CQ, a lysosomal inhibitor that blocks autophagosome–lysosome fusion and cargo degradation. Co‐treatment with CQ (20 μm) and ML‐SA5 resulted in a significant accumulation of both LC3II and p62 compared to ML‐SA5 treatment alone (Fig. S2). This CQ‐dependent accumulation confirms that active autophagic degradation is ongoing in ML‐SA5‐treated cells. These findings demonstrate that ML‐SA5 enhances autophagosome formation and restores fusion with lysosomes, thereby promoting autophagic flux (Fig. 3H,I).

### ML‐SA5 reverses TGF‐β1‐induced activation of RPLFs and AMLFs

**Fig. 4:**
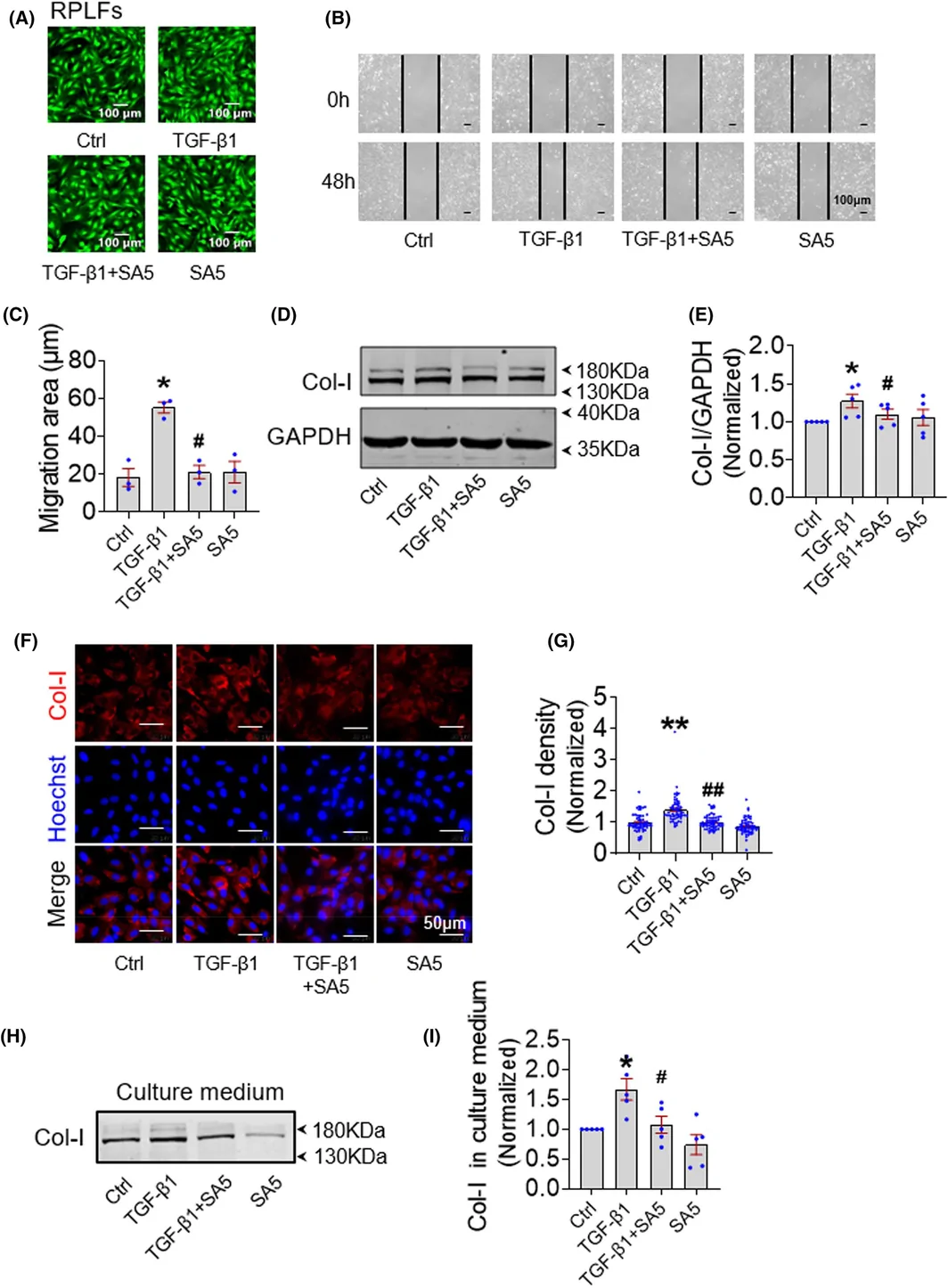
ML‐SA5 reverses transforming growth factor‐β1 (TGF‐β1)‐induced activation and modulates autophagy in rat primary lung fibroblasts (RPLFs). RPLFs were divided into four groups: control (Ctrl), TGF‐β1 alone (10 ng/mL), TGF‐β1 combined with ML‐SA5 (1 μm), and ML‐SA5 alone (1 μm). ML‐SA5 was added 30 min prior to TGF‐β1 stimulation, and cells were harvested after 48 h. (A) Representative morphology of RPLF cells under light microscopy and fluorescence microscopy following Calcein‐AM staining. Scale bar = 100 μm. (B) Representative images of wound‐healing assay in RPLFs at 0 and 48 h post‐treatment. (C) Quantitative analysis of migration distance within 48 h. *P < 0.05 vs. Ctrl, #*P* < 0.05, vs. TGF‐β1, *n* = 3. (D, E) Representative immunoblots and quantification of collagen I (Col‐I) expression in RPLF cells. *P < 0.05 vs. Ctrl, #*P* < 0.05, vs. TGF‐β1, *n* = 5. (F, G) Representative immunofluorescence images and quantification of Col‐I staining in RPLF cells. Red, Col‐I; blue, nuclei. Scale bar = 50 μm. **P < 0.01 vs. Ctrl; ##*P* < 0.01 vs. TGF‐β1, *n* = 30 cells from 6 independent biological replicates. (H, I) Collagen expression in the supernatant of RPLFs. *P < 0.05 vs. Ctrl, #*P* < 0.05, *n* = 5. Data are presented as mean ± SEM. One‐way ANOVA followed by Tukey's *post hoc* test and Kruskal–Wallis test for one‐way ANOVA followed by the Dunn *post hoc* test were used for statistical analysis.

**Fig. 5:**
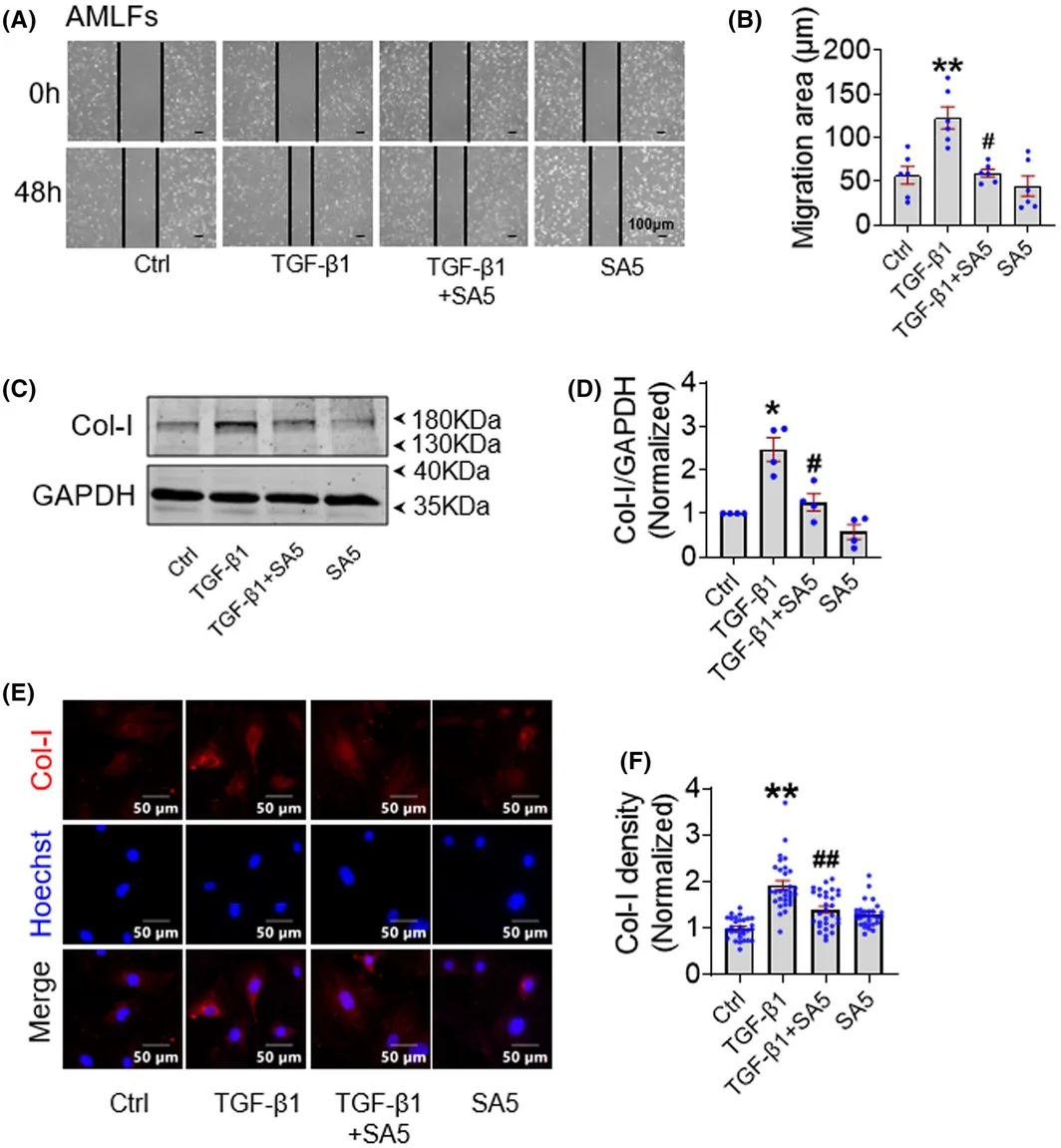
ML‐SA5 reverses transforming growth factor‐β1 (TGF‐β1)‐induced activation of adult mouse lung fibroblasts (AMLFs). (A, B) Representative images of wound‐healing assays in AMLFs at 0 and 48 h, along with quantitative analysis of the migration distance. **P < 0.01 vs. Ctrl; #*P* < 0.05, *n* = 6. (C, D) Protein expression levels and quantitative analysis of collagen I (Col‐I) in AMLFs cells. *P < 0.05 vs. Ctrl; #*P* < 0.05, *n* = 4. (E, F) Representative immunofluorescence images and quantification of Col‐I expression in AMLFs cells. **P < 0.01 vs. Ctrl, ##*P* < 0.01, vs. TGF‐β1, *n* = 30 cells from 6 independent biological replicates. Data are presented as mean ± SEM. One‐way ANOVA followed by Tukey's *post hoc* test and Kruskal–Wallis test for one‐way ANOVA followed by the Dunn *post hoc* test were used for statistical analysis.

To validate these findings in primary cells, we examined ML‐SA5 effects in RPLFs. Calcein‐AM staining showed that TGF‐β1 induced a denser, more robust morphology compared with spindle‐shaped controls; 1 μm ML‐SA5 reversed these morphological changes (Fig. 4A). Wound‐healing assays confirmed that TGF‐β1‐enhanced RPLF migration was significantly inhibited by ML‐SA5 (Fig. 4B,C). Western blot and immunofluorescence analyses demonstrated that ML‐SA5 attenuated TGF‐β1‐induced Col‐I accumulation in RPLFs, consistent with results obtained in MRC‐5 cells (Fig. 4D–G). Furthermore, we have also examined collagen I protein expression in the conditioned medium of RPLFs. This measurement is commonly used in the fibrosis field in previous studies [29, 30]. Our results showed that collagen I in culture medium is increased up to 60–70% compared with the control group, which is reversed after ML‐SA5 treatment (Fig. 4H,I). Furthermore, we have also isolated and cultured AMLFs and validated our findings in these primary adult cells. Consistently, the results from these AMLFs recapitulated our original findings: pharmacological modulation of TRPML1 activity produced functional effects on cell migration (Fig. 5A,B) and collagen I expression (Fig. 5C–F) that were entirely consistent with our observations in MRC‐5 cells and RPLFs.

### ML‐SA5 exerts anti‐fibrotic effects through mTOR‐dependent autophagy modulation

**Fig. 6:**
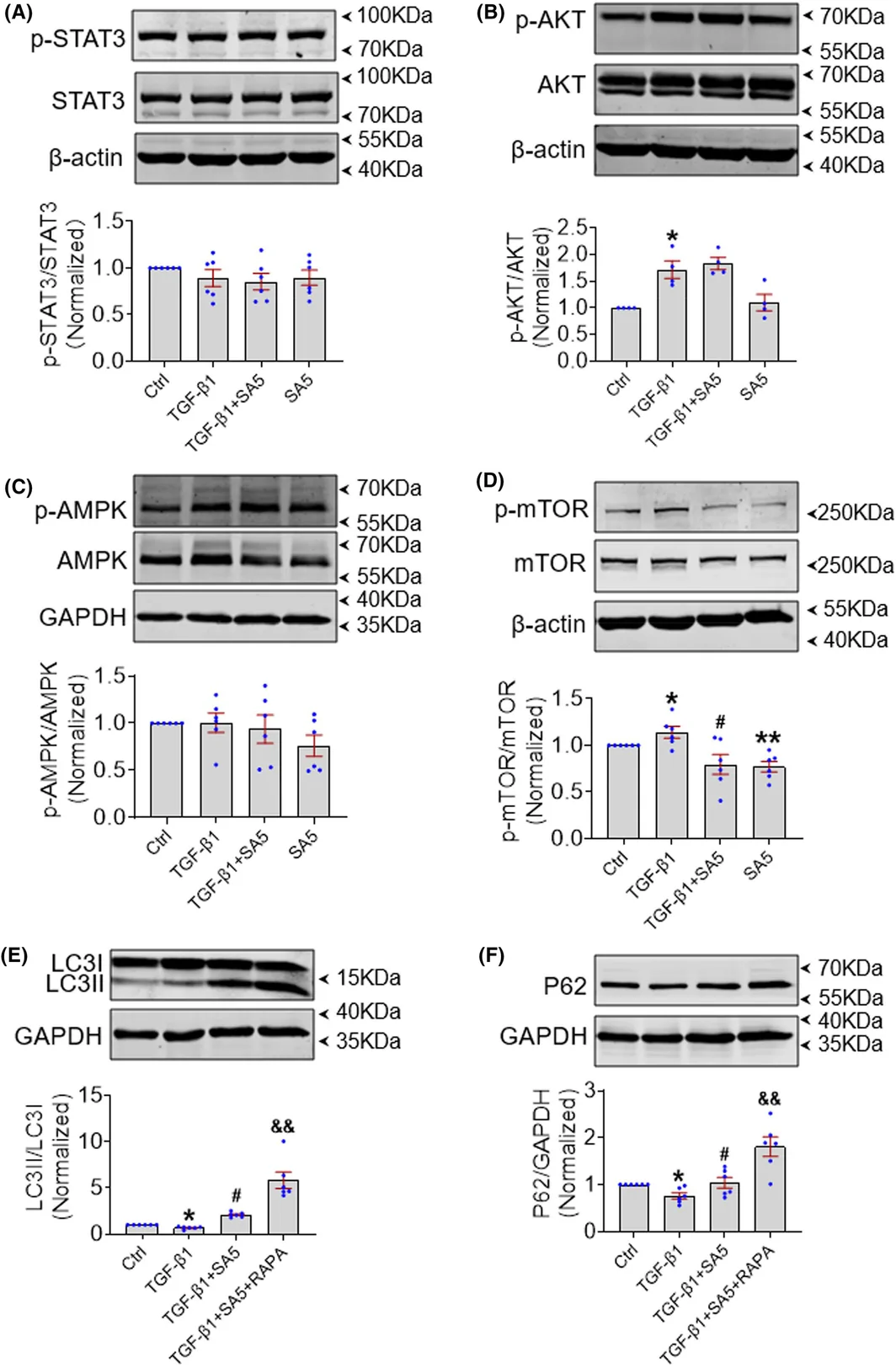
ML‐SA5 exerts anti‐fibrotic effects through mTOR‐dependent autophagy modulation. MRC‐5 cells were divided into four groups: control (Ctrl), TGF‐β1 alone (10 ng/mL), TGF‐β1 combined with ML‐SA5 (1 μm), and ML‐SA5 alone (1 μm). ML‐SA5 was added 30 min prior to TGF‐β1 stimulation, and cells were harvested after 48 h. (A, B) Representative immunoblot images and protein expression of p‐signal transducer and activator of transcription 3 (STAT3)/STAT3 (*n* = 6), p‐AKT/AKT (*n* = 4) in MRC‐5 cells. *P < 0.05 vs. Ctrl. (C, D) Representative immunoblot images and protein expression of p‐adenosine 5′‐monophosphate‐activated protein kinase (AMPK)/AMPK (*n* = 6) and p‐mammalian target of rapamycin (mTOR)/mTOR (*n* = 6) in MRC‐5 cells. *P < 0.05, **P < 0.01 vs. Ctrl, #*P* < 0.05 vs. TGF‐β1. To clarify mTOR involvement, we used the mTOR inhibitor rapamycin (RAPA). MRC‐5 cells received one of four treatments: control, TGF‐β1 (10 ng/mL) alone, TGF‐β1 + ML‐SA5 (1 μm), or TGF‐β1 + ML‐SA5 + RAPA (10 μm). The sequential regimen was RAPA for 30 min, followed by ML‐SA5 for 30 min, then TGF‐β1 stimulation; cells were collected 48 h after TGF‐β1 addition. (E, F) Protein expression of autophagy markers in MRC‐5 cells treated with ML‐SA5 alone or in combination with RAPA. *P < 0.05, #*P* < 0.05 vs. TGF‐β1; &&*P* < 0.01 vs. TGF‐β1 + SA5, *n* = 6. Data are presented as mean ± SEM. Kruskal–Wallis test for one‐way ANOVA followed by the Dunn *post hoc* test was used for statistical analysis.

To identify signaling pathways mediating the anti‐fibrotic effects of ML‐SA5, we assessed several candidate kinases. ML‐SA5 did not significantly alter phosphorylation of STAT3, AKT, or AMPK. In contrast, ML‐SA5 significantly reduced mTOR phosphorylation, implicating mTOR inhibition as a potential mechanism underlying ML‐SA5‐induced autophagy and anti‐fibrotic activity (Fig. 6A–D). To confirm the causal role of mTOR in ML‐SA5‐mediated anti‐fibrotic and autophagy‐modulating effects, we performed co‐treatment experiments with RAPA (a classic mTOR inhibitor). Combined treatment with ML‐SA5 and rapamycin produced a greater increase in the LC3II/LC3I ratio and p62 expression than ML‐SA5 alone, further supporting mTOR‐dependent autophagy activation (Fig. 6E,F).

### Modulation of mTOR activity regulates the anti‐pulmonary fibrotic effects of ML‐SA5

**Fig. 7:**
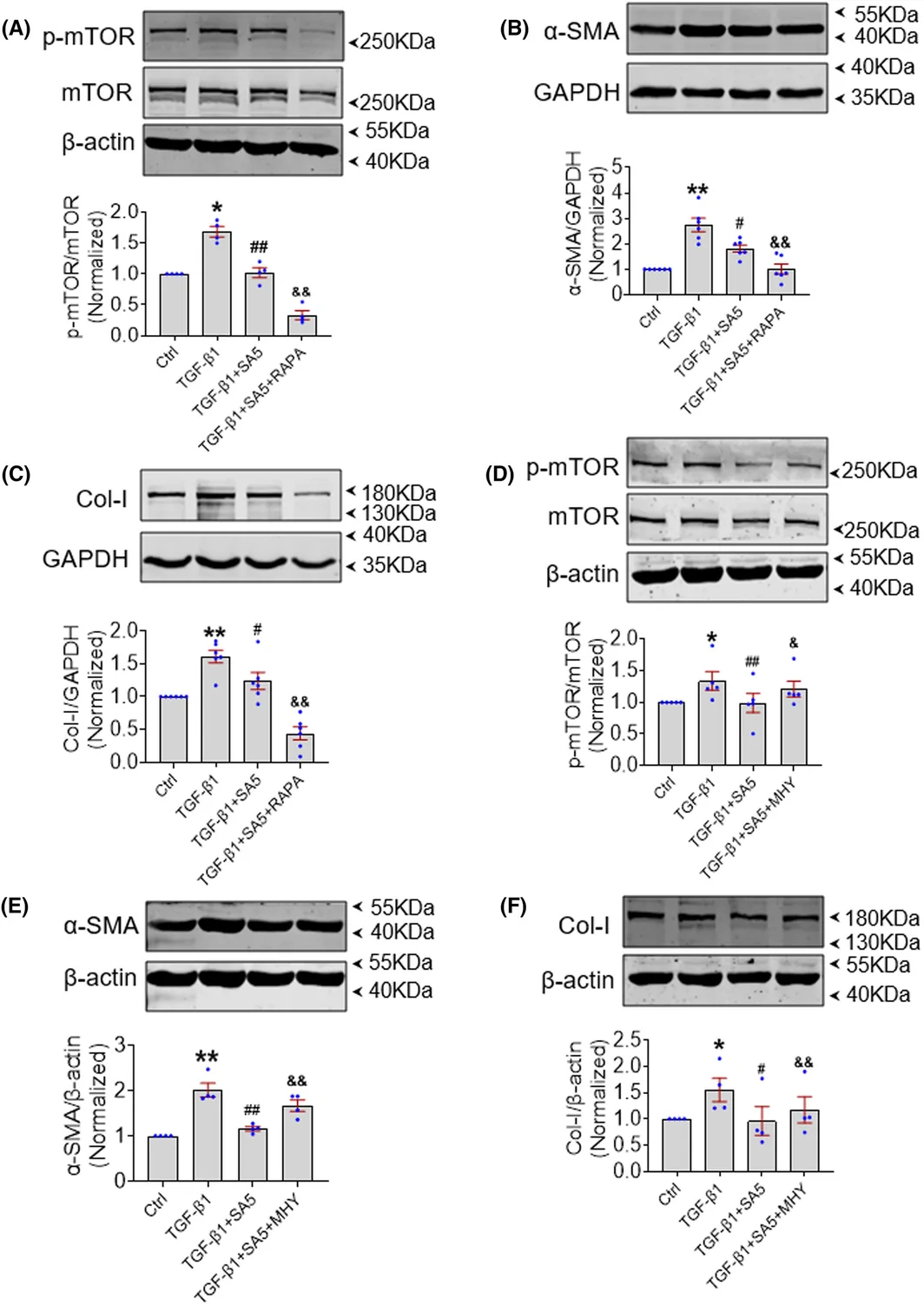
Modulation of mammalian target of rapamycin (mTOR) activity regulates the anti‐pulmonary fibrotic effects of ML‐SA5. To investigate the involvement of mTOR, the inhibitor rapamycin (RAPA) and the activator MHY1485 (MHY) were used. MRC‐5 cells were divided into the following groups: Control, TGF‐β1 (10 ng/mL) alone, TGF‐β1 + ML‐SA5 (1 μm), TGF‐β1 + ML‐SA5 + RAPA (10 μm), or TGF‐β1 + ML‐SA5 + MHY (2 μm). For the combined groups, RAPA or MHY was added 30 min prior to ML‐SA5, which was in turn added 30 min before TGF‐β1 stimulation; all cells were harvested 48 h post‐stimulation. (A–C) Protein expression of p‐mTOR/mTOR (*n* = 4) and fibrotic markers α‐smooth muscle Actin (α‐SMA, *n* = 6) and collagen I (Col‐I, *n* = 6) in MRC‐5 cells treated with ML‐SA5 alone or in combination with rapamycin. *P < 0.05, **P < 0.01 vs. Ctrl; #*P* < 0.05, ##*P* < 0.01 vs. TGF‐β1; &&*P* < 0.01 vs. TGF‐β1 + SA5. (D‐F) Protein expression of p‐mTOR/mTOR (n = 5) and fibrotic markers α‐SMA and Col‐I (*n* = 4) in MRC‐5 cells treated with ML‐SA5 alone or in combination with MHY1485. *P < 0.05, **P < 0.01 vs. Ctrl; #*P* < 0.05, ##*P* < 0.01 vs. TGF‐β1; &*P* < 0.05, &&*P* < 0.01 vs. TGF‐β1 + SA5. Data are presented as mean ± SEM. Kruskal–Wallis test for one‐way ANOVA followed by the Dunn *post hoc* test was used for statistical analysis.

To further validate the role of mTOR in ML‐SA5‐mediated anti‐fibrosis, we performed co‐treatment experiments with RAPA or MHY1485 (a selective mTOR activator). Combined treatment with ML‐SA5 and rapamycin elicited enhanced inhibition of p‐mTOR, Col‐I, and α‐SMA (Fig. 7A–D). Conversely, co‐treatment with MHY1485 increased p‐mTOR levels and reversed ML‐SA5‐mediated suppression of Col‐I and α‐SMA (Fig. 7E–H). Collectively, these findings confirm that the modulation of mTOR activity directly regulates the anti‐pulmonary fibrotic effects of ML‐SA5 and further support that mTOR inhibition is a key mechanism underlying the anti‐fibrotic action of ML‐SA5.

## Discussion

PF is a devastating disease characterized by progressive deposition of extracellular matrix components, particularly type I collagen, within the lung parenchyma. Its pathogenesis involves complex intercellular interactions, and effective therapeutic interventions remain limited [31, 32]. This study investigated the mechanism of action of ML‐SA5, a selective agonist of the lysosomal cation channel TRPML1, in TGF‐β1‐induced lung fibroblasts. Our results demonstrate that ML‐SA5 significantly attenuated TGF‐β1‐induced morphological changes and migratory capacity in both MRC‐5 cells and RPLFs. Moreover, ML‐SA5 effectively suppressed TGF‐β1‐induced upregulation of fibrotic markers, including α‐SMA, Col‐I, CTGF, and TGF‐β1 itself. Through analysis of LC3, p62, and autophagic flux dynamics, we found that ML‐SA5 attenuates fibroblast activation by restoring autophagic flux. Mechanistically, ML‐SA5 promoted autophagy through inhibition of mTOR phosphorylation, thereby enhancing lysosomal degradation of ECM components. These effects were reversed by the mTOR activator MHY1485, confirming mTOR dependence.

TRPML1, a non‐selective cation channel localized to lysosomal membranes, plays a crucial role in lysosomal Ca^2+^ release and maintenance of lysosomal homeostasis [12]. It participates in diverse cellular processes, including autophagy–lysosome pathway regulation, membrane trafficking, and signal transduction [13, 14]. In neurodegenerative diseases and lysosomal storage disorders, TRPML1 dysfunction is closely associated with impaired autophagic flux and aberrant protein aggregation [33, 34]. However, research on its role in fibrotic diseases remains relatively scarce [15, 16]. Our results are consistent with the recent study by Weiden et al., who demonstrated that TRPML1 suppresses PF by limiting collagen and elastin deposition through increasing matrix metalloproteinase levels [17]. Both studies support the concept that pharmacological activation of TRPML1 represents a promising anti‐fibrotic strategy. Previous studies demonstrated that loss or blockade of TRPML1 channel reduced autophagy [35, 36, 37]. In contrast, Weiden et al. further observed that the intracellular collagen was unchanged between WT and TRPML1‐deficient lung fibroblasts. However, the regulatory mechanisms underlying TRPML1 and autophagy in PF remain not fully understood. Our present study mainly focused on this point. Notably, several important distinctions exist between the two studies. First, while Weiden et al. focused primarily on the general anti‐fibrotic effects of TRPML1 agonists in PF models, our study specifically elucidates the mTOR‐dependent mechanism underlying ML‐SA5‐mediated autophagy restoration in lung fibroblasts. Second, we provide direct evidence that ML‐SA5 inhibits mTOR phosphorylation, and that this inhibition is both necessary and sufficient for the observed anti‐fibrotic effects, as demonstrated by rescue experiments using the mTOR activator MHY1485. Third, our use of the mCherry‐GFP‐LC3 tandem reporter system provides dynamic assessment of autophagic flux, complementing the static marker analyses performed by Weiden et al. Together, these studies collectively strengthen the rationale for targeting TRPML1 in PF, while our work adds mechanistic depth by identifying mTOR as a critical signaling node in the TRPML1–autophagy axis. TRPML1 protein expression was significantly downregulated in TGF‐β1‐stimulated lung fibroblasts (Fig. 1A) and in bleomycin‐induced fibrotic lung tissues compared with the sham controls (Fig. S3). Pharmacological activation with ML‐SA5 restored TRPML1 expression and reversed the fibrotic phenotype, functionally linking TRPML1 to autophagy regulation in PF and offering new insights into fibrotic disease pathogenesis. Notably, the ML‐SA5 concentrations employed in this study (1–10 μm) align with literature‐reported doses that specifically amplify TRPML1 currents and activate downstream signaling [18, 19, 35].

During PF progression, autophagic status varies distinctly among different cell types. In damaged alveolar epithelial cells, autophagic activity is often suppressed, leading to accumulation of damaged mitochondria, excessive reactive oxygen species (ROS) production, and activation of the NLRP3 inflammasome, which drives secretion of pro‐fibrotic factors [38, 39]. Conversely, activated fibroblasts may exhibit enhanced autophagy to support proliferation, resistance to apoptosis, and ECM synthesis. Our results indicate that TGF‐β1 suppressed autophagy markers in MRC‐5 cells, whereas ML‐SA5 restored autophagic flux and promoted autophagosome–lysosome fusion. Importantly, dynamic autophagic flux analysis using the mCherry‐GFP‐LC3 tandem reporter system demonstrated that ML‐SA5 increased functional autolysosome formation, indicating that restoration of impaired autophagic function—rather than directionality of specific markers—correlates with anti‐fibrotic efficacy. We acknowledge that the interpretation of p62 as an autophagy marker requires caution. Although p62 is commonly regarded as an autophagic substrate whose steady‐state levels inversely correlate with autophagic activity [40], its expression is also subject to complex transcriptional regulation by stress‐responsive transcription factors such as NRF2 and TFEB, which can drive compensatory upregulation during autophagy activation [41]. In our study, TGF‐β1 stimulation reduced p62 protein levels, whereas ML‐SA5 restored p62 expression concurrent with increased LC3II and functional autolysosome formation. To rule out the possibility that elevated p62 reflected impaired lysosomal degradation rather than active flux, we performed CQ blockade experiments. Co‐treatment with CQ and ML‐SA5 resulted in further accumulation of both LC3II and p62 compared with ML‐SA5 alone, confirming that active autophagic degradation was ongoing and that the lysosomal pathway remained functional. Thus, the elevated p62 observed following ML‐SA5 treatment most likely represents a dynamic balance between increased *de novo* synthesis and active autophagic turnover, rather than a block in autophagic clearance. Collectively, our conclusions regarding autophagic flux restoration are grounded in the direct visualization of autophagosome‐to‐autolysosome maturation via the mCherry‐GFP‐LC3 reporter system, with p62 and LC3II data serving as complementary, but not standalone, indicators of autophagic status.

RAPA has been shown to reduce collagen deposition and α‐SMA expression, improving lung tissue structure in experimental fibrosis models [42, 43]. Our study demonstrated that combined treatment with ML‐SA5 and RAPA produced synergistic enhancement of autophagic flux and further suppression of fibrotic markers, reinforcing the critical role of mTOR inhibition in this process. Conversely, the mTOR activator MHY1485, which directly stimulates mTORC1 signaling, effectively reversed both ML‐SA5‐induced autophagy activation and subsequent Col‐I inhibition. These findings not only establish mTOR as an upstream mediator of ML‐SA5 effects but also provide a theoretical rationale for combination therapeutic strategies targeting the TRPML1‐mTOR‐autophagy axis. The observation that ML‐SA5 inhibits mTOR phosphorylation without affecting Akt (Ser473) phosphorylation is noteworthy. Akt Ser473 phosphorylation is primarily mediated by mTORC2 and is required for full Akt activation [44]. The dissociation between mTORC1 inhibition (reduced p‐mTOR) and intact mTORC2 signaling (unchanged p‐Akt Ser473) suggests that ML‐SA5 selectively targets mTORC1, which is the primary regulator of autophagy initiation.

Our finding that ML‐SA5 restores autophagic flux through mTOR inhibition adds a new layer to the current understanding of TRPML1‐mediated autophagy regulation. Notably, a recent study by Cunha et al. demonstrated that the earlier‐generation TRPML1 agonist ML‐SA1 promotes autophagy via the Ca^2+^‐calcineurin‐TFEB axis, leading to transcriptional upregulation of CLEAR‐network genes [45]. Although we used ML‐SA5 instead of ML‐SA1 – primarily because of its substantially higher potency and superior aqueous solubility [18, 19], which allow for more reliable mechanistic interrogation at lower concentrations – our results are not contradictory but rather complementary to that work. Together, these studies suggest that TRPML1 activation can engage at least two distinct but potentially synergistic signaling routes: a transcription‐dependent pathway through TFEB and a post‐translational, rapid pathway through mTORC1 inhibition. Given that mTORC1 is anchored on the lysosomal surface and its activity is modulated by lysosomal Ca^2+^ signals, it is plausible that TRPML1‐mediated Ca^2+^ release simultaneously influences both calcineurin‐TFEB and mTORC1, depending on cell type and pathological context. Thus, our work, together with that of Cunha et al., expands the mechanistic repertoire of TRPML1 in autophagy regulation and underscores the potential of TRPML1 agonists as versatile tools for restoring autophagic balance in fibrotic and other lysosome‐related disorders.

Targeting TRPML1 with selective agonists offers several theoretical advantages for PF treatment. First, lysosome‐directed therapy may potentially achieve tissue‐specific effects with reduced systemic exposure. Second, modulating autophagic flux—rather than simply activating or inhibiting autophagy—represents a more nuanced physiological intervention. Third, targeting non‐Smad pathway mechanisms may circumvent resistance associated with direct TGF‐β inhibition. Future research should explore combinatorial effects of ML‐SA5 with existing anti‐fibrotic agents such as nintedanib and pirfenidone [46], and focus on developing next‐generation TRPML1 modulators with optimized selectivity and pharmacokinetic properties.

Several limitations warrant acknowledgment. First, it should be noted that our study did not include adult primary human lung fibroblasts, which would represent the most clinically relevant model. However, the consistency of our results across MRC‐5 cells, neonatal rat, and adult mouse fibroblasts suggests that the observed effects are not cell‐line‐ or species‐specific. Nonetheless, validation in adult primary human fibroblasts would further strengthen the clinical implications of our findings. Second, pharmacological tools have inherent specificity limitations; future studies should employ genetic gain‐ or loss‐of‐function approaches to validate these findings.

In conclusion, this study systematically elucidates the molecular mechanism by which the TRPML1 agonist ML‐SA5 exerts anti‐fibrotic effects through mTOR inhibition and restoration of autophagic flux. These findings establish a novel role for the lysosome‐autophagy pathway in PF pathogenesis and provide experimental support for therapeutic strategies targeting TRPML1. Subsequent studies should validate ML‐SA5 efficacy and safety in animal models of pulmonary fibrosis, develop highly selective TRPML1 modulators, explore combination regimens with existing anti‐fibrotic drugs, and evaluate the broader applicability of this approach to fibrotic diseases of other organs. Together, these findings suggest that pharmacological activation of TRPML1 to restore autophagic flux in lung fibroblasts represents a promising therapeutic strategy for PF.

## Author contributions

JZ, PH, and HZ: Conceptualization, Supervision, Writing‐Original draft preparation, Writing‐Reviewing and Editing; JY, XK, LM, SZ, QL, JZ, MX, and CL: Methodology, Investigation. All authors approved the submission of this manuscript.

## Supporting information


**Fig S1.** Effects of the TRPML1 agonist ML‐SA5 on intracellular Ca^2+^ concentration in rat primary lung fibroblasts (RPLFs). (A) Representative Fluo‐8/AM fluorescence images of RPLFs at baseline, after 10 min of TRPML1 agonist ML‐SA5 (SA5, 1 μm) treatment, and following a subsequent 10‐min incubation with the antagonist ML‐SI3 (SI3, 2.5 μm). (B) Quantification of relative fluorescence intensities. Data are shown as means ± SEM. **P < 0.01 vs. control; ##*P* < 0.01 vs. ML‐SA5‐treated group; *n* = 30 cells per group. Statistical analysis was performed using one‐way ANOVA followed by Tukey's *post hoc* test.
**Fig. S2.** Effects of the lysosomal inhibitor chloroquine (CQ) on LC3 and p62 protein levels in TGF‐β1‐treated MRC‐5 cells. MRC‐5 cells were treated with TGF‐β1 (10 ng/mL) and/or ML‐SA5 (1 μm) in the presence or absence of CQ (20 μm) for 48 h. (A) Representative western blot bands of LC3. (B) Densitometric quantification of the LC3II/I ratio. (C) Representative western blot bands of p62. (D) Densitometric quantification of p62. Data are shown as means ± SEM. *P < 0.05 vs. control; #*P* < 0.05 vs. TGF‐β1 group; &*P* < 0.05, &&*P* < 0.01 vs. TGF‐β1 + ML‐SA5 group, n = 6. Statistical analysis was performed using the Kruskal–Wallis test followed by Dunn's *post hoc* test.
**Fig. S3.** TRPML1 protein expression in lung tissues from a bleomycin‐induced mouse model of pulmonary fibrosis. (A) Representative western blot bands of TRPML1 in lung tissues from fibrotic mice versus sham controls (model established as described in Basic Clin Pharmacol Toxicol. 2026;138(1):e70158). (B) Densitometric quantification of TRPML1 expression. Data are means ± SEM. **P < 0.01 vs. sham; *n* = 6 mice per group; each lane represents an individual mouse (biological replicate). Western blotting was performed once for each sample, with GAPDH as a loading control. Statistical analysis was performed using the unpaired Student's *t*‐test.

## Data Availability

The data that support the findings of this study are available from the corresponding author upon reasonable request.
