# Supplementary material for: TRPML1 agonist ML‐SA5 attenuates pulmonary fibroblast activation by suppressing mTOR and restoring autophagic flux
Source: FEBS Open Bio. 2026 Sep 24:10.1002/2211-5463.70346. Online ahead of print. doi: 10.1002/2211-5463.70346 (PMC13602826; doi:10.1002/2211-5463.70346)
Supplement: Supplementary file 1 [file FEB4-9999-0-s001.docx]

**Supplementary data**

**Fig. S1. Effects of the TRPML1 agonist ML-SA5 on intracellular Ca^2+^ concentration in rat primary lung fibroblasts (RPLFs).** (A) Representative Fluo-8/AM fluorescence images of RPLFs at baseline, after 10 min of TRPML1 agonist ML-SA5 (SA5, 1 μM) treatment, and following a subsequent 10-min incubation with the antagonist ML-SI3 (SI3, 2.5 μM). (B) Quantification of relative fluorescence intensities. Data are shown as means ± SEM. ***P* < 0.01 vs. control; ##*P* < 0.01 vs. ML-SA5-treated group; n = 30 cells per group. Statistical analysis was performed using one-way ANOVA followed by Tukey's post hoc test.

**Fig. S2. Effects of the lysosomal inhibitor chloroquine (CQ) on LC3 and p62 protein levels in **TGF-β1-treated MRC-5 cells.** MRC-5 cells were treated with** TGF-β1 (10 ng/mL) and/or ML-SA5 (1 μM) in the presence or absence of CQ (20 μM) for 48 h. **(A)** Representative Western blot bands of LC3. (B) Densitometric quantification of the LC3II/I ratio. **(C)** Representative Western blot bands of p62. (D) Densitometric quantification of p62. Data are shown as means ± SEM. **P* < 0.05 vs. control; #*P* < 0.05 vs. TGF-β1 group; &*P* < 0.05, &&*P* < 0.01 vs. TGF-β1+ML-SA5 group, n = 6. Statistical analysis was performed using the Kruskal-Wallis test followed by Dunn’s post hoc test.

**Fig. S3. TRPML1 protein expression in lung tissues from a bleomycin-induced mouse model of pulmonary fibrosis.** (A) Representative Western blot bands of TRPML1 in lung tissues from fibrotic mice versus sham controls (model established as described in Basic Clin Pharmacol Toxicol. 2026;138(1):e70158). (B) Densitometric quantification of TRPML1 expression. Data are means ± SEM. ***P* < 0.01 vs. sham; n = 6 mice per group; each lane represents an individual mouse (biological replicate). Western blotting was performed once for each sample, with GAPDH as a loading control. Statistical analysis was performed using the unpaired Student's t-test.
